# Environmental Stressors and Intimate Partner Violence in Urban Tanzania: A Thematic and Visual Analysis from Dar es Salaam

**DOI:** 10.3390/ijerph23020204

**Published:** 2026-02-05

**Authors:** Deo Mshigeni, Salome Kapella Mshigeni

**Affiliations:** 1 Division of Family & Culture, College of Behavioral and Social Sciences, California Baptist University, Riverside, CA 92504, USA; dmshigeni@calbaptist.edu; 2 Department of Health Science and Human Ecology, College of Natural Sciences, California State University, San Bernardino, CA 92407, USA

**Keywords:** health disparities, discrimination, health, gender inequality, determinants

## Abstract

Background: This study explores the interplay between environmental stressors and intimate partner violence (IPV) in Dar es Salaam, Tanzania. Methods: Drawing on participants’ interviews, visual ethnography, thematic analysis, and a review of secondary sources, the research examines how resource scarcity, displacement, and climate change intersect with social determinants of health to intensify IPV. Results: Using an ecological systems perspective, the study demonstrates how structural vulnerabilities and environmental degradation disproportionately affect residents of informal urban settlements, particularly women, who face intersecting vulnerabilities due to poverty, inadequate services, and gender-based discrimination. Conclusions: The findings from this study underscore the need to integrate gender-sensitive urban planning and policy that address both environmental risks and existing social inequalities, thereby enhancing household and community resilience.

## 1. Introduction

Environmental stressors such as resource scarcity and climate change increasingly shape the well-being of urban households, particularly in informal settlements [1,2]. These stressors, compounded by displacement, unemployment, and inadequate infrastructure, can lead to domestic instability and increased rates of Intimate Partner Violence (IPV). This study explores the relationship between environmental stress and IPV in urban Dar es Salaam, Tanzania, using a multidisciplinary approach. The research integrates a multicultural feminist perspective with ecological and public health frameworks to understand the specific vulnerabilities faced by women and children [3,4].

While generally, domestic violence is often discussed through psychological or criminological lenses, this paper situates IPV within broader environmental, socio-economic, and cultural dynamics. In doing so, we underscore the importance of addressing both proximate and structural causes of gender-based violence in urban contexts, particularly in Tanzania. According to [5], the normalization of IPV in some Sub-Saharan African communities heightens health risks and perpetuates intergenerational harm. Therefore, a multi-scalar analysis that connects place, policy, and lived experience is needed to address the limitations of current, often fragmented, interventions that fail to integrate environmental, gender, and urban planning dimensions. Building on this perspective, the case of urban Dar es Salaam illustrates how rapid urbanization, spatial inequality, and socio-economic precarity intersect to intensify IPV. As Tanzania’s largest city, Dar es Salaam is marked by informal settlements, overcrowded housing, and limited access to social services, all of which create environments where IPV can be both hidden and normalized. Gendered expectations, economic stress, and weak enforcement of protective laws compound the vulnerability of women in low-income neighborhoods. Scholarships focusing on urban East Africa point to women in informal urban settings facing higher risks of IPV due to constrained mobility, economic dependence, and limited institutional recourse [6,7]. Thus, any intervention strategy must consider not only individual behaviors but also the structural urban dynamics that shape everyday experiences of violence.

### 1.1. Background and Context

Intimate Partner Violence is commonly defined as a pattern of abusive behavior used by one partner to exert control over another in an intimate relationship [8]. While widely used definitions of IPV are comprehensive, they often emphasize patterns of control and coercion, potentially overlooking episodic or situational forms of violence exacerbated by environmental and economic stress [9]. The World Health Organization (WHO) expands the definition to include physical violence, sexual violence, emotional abuse, and controlling behaviors by a current or former partner [10]. Similarly, the Centers for Disease Control and Prevention (CDC) describe IPV as encompassing a range of behaviors, physical violence, sexual violence, stalking, and psychological aggression that can occur in both ongoing and terminated relationships [11]. Also, IPV may also occur in isolated incidents, stem from mutual conflict, or be influenced by mental illness, trauma, or intoxication [9]. Not all IPV follows a pattern of coercive control; in some cases, it arises situationally, triggered by external stressors such as displacement, trauma, or poverty [12,13].

Globally, women experience IPV at significantly higher rates than men, with one in four women reporting physical or sexual violence by a partner since the age of 15 [9,14]. These acts of violence have far-reaching consequences for individual well-being, child development, public health, and community safety [15,16]. Although IPV affects all social strata, structural inequalities such as poverty, discrimination, and limited access to social support exacerbate women’s risk [9,17].

In urban contexts, such as Dar es Salaam, environmental hazards and infrastructural deficits intersect with social stressors to heighten tensions within households [18]. Poor infrastructure, overcrowding, and recurring climate-related disruptions, such as flooding and drought, place families under stress. These conditions, compounded by unemployment, displacement, and lack of services, form a volatile ecosystem in which IPV is more likely to occur [19]. Understanding how environmental and social systems interact to affect IPV requires moving beyond traditional risk factors to include broader ecological conditions.

### 1.2. The Interplay of Environmental Stressors and Intimate Partner Violence

Environmental stressors such as climate change, resource scarcity, and displacement disrupt household dynamics in urban contexts, escalating the risk of IPV in most low-income communities. For example, in areas with already poor infrastructure, such as Dar es Salaam, these stressors heighten household tension, reduce coping capacities, and undermine social cohesion [20,21]. Furthermore, events such as droughts, floods, or urban heatwaves can destroy livelihoods or displace populations, generating social instability and exacerbating existing inequalities [22,23].

In urban Dar es Salaam, such dynamics are increasingly visible, shaping daily stressors that disproportionately affect women and low-income households, with implications for heightened vulnerability to interpersonal violence and community safety concerns. For instance, recurrent flooding in informal settlements like Kigogo and Jangwani continuously displaces families and damages property, often forcing households into temporary shelters or overcrowded housing with minimal privacy. These abrupt shifts in living conditions, combined with loss of income due to damage to informal businesses or street vending, can increase stress within households and create conditions in which women are at increased risk of experiencing IPV, due to systemic failures that enable perpetration and limit recourse. For example, following the heavy floods of 2020, women’s rights groups in Dar es Salaam reported a spike in IPV cases, particularly in flood-prone wards where infrastructure is weak and public services are scarce [24,25,26]. Additionally, prolonged water shortages in neighborhoods such as Buguruni and Mbagala have led to increased domestic tension, as women, who are primarily responsible for fetching water, face long queues and confrontations, often returning home late. This, in turn, can mislead some partners into interpreting these delays as signs of marital infidelity, thereby fueling suspicion and conflict within households. These examples underscore how environmental pressures interact with gender roles and socio-economic marginalization to exacerbate IPV in the urban fabric of Dar es Salaam.

Further, these environmental stressors do not act in isolation; their impact is deeply mediated by social determinants of health (SDOH) such as poverty, unemployment, low education, gender inequality, and inadequate access to essential services [27,28]. In the city of Dar es Salaam, these intersecting factors are especially pronounced in low-income areas such as Tandale, Kigogo, Vingunguti, Buguruni, Msimbazi, and Temeke. In these specific areas, high rates of unemployment, particularly among youth and men, create economic pressures that strain household relationships. When coupled with environmental disruptions such as flooding or prolonged water shortages, tensions escalate, and the risk of IPV within the household ultimately increases.

For example, in the densely populated settlement of Tandale, women often rely on petty trade or informal labor, which is highly sensitive to environmental shocks. When market stalls are destroyed during seasonal floods, as happened in early 2023, many women lost their livelihoods overnight. Without safety nets or access to credit, economic dependency on male partners deepens, reducing women’s autonomy and ability to leave unsafe relationships, access justice systems, or secure independent livelihoods—factors that in turn heighten the risk of sustained abuse [29]. Further, in neighborhoods like Buguruni, where public services are limited and education levels are low, access to information about rights and protective services is minimal. As a result, women facing IPV often remain silent due to fear, stigma, or lack of alternatives. These real-time examples from Dar es Salaam illustrate how environmental challenges intersect with entrenched social inequalities to compound the risk of IPV in urban settings.

Building on these lived realities in Dar es Salaam, the model of family interdependence and ecological systems theory helps frame how stressors at multiple levels, from insecure housing to policy neglect, intersect with personal dynamics to shape the risk of IPV. While violence is ultimately a choice made by perpetrators, broader pressures may exacerbate patterns of control or escalation [30,31]. These frameworks emphasize that violence within households does not arise in a vacuum but is embedded within overlapping layers of stressors from the individual to the societal level. When men in neighborhoods like Tandale or Buguruni face chronic unemployment or loss of status due to economic disruptions, or when women are overwhelmed with caregiving responsibilities in unstable and under-resourced environments, the resulting psychological strain often manifests in heightened household conflict. In these conditions, IPV may be both a symptom of individual distress and a reflection of deeper systemic breakdowns. As such, a comprehensive IPV response must address perpetration directly, while also recognizing how structural factors, such as housing insecurity, climate shocks, and social exclusion, limit women’s ability to seek safety or justice, which in turn intensifies household stress and reduces resilience in urban settings like Dar es Salaam.

## 2. Literature Review

Intimate partner violence remains a global concern, affecting individuals across diverse socio-economic backgrounds and cultures [32]. The literature suggests that IPV includes a range of behaviors, physical, sexual, emotional, psychological, and financial abuse, often perpetrated by intimate partners or family members [10]. Moreover, its impacts are both immediate and long-term, affecting mental health, physical safety, social mobility, and child development [33,34]. For example, research shows that children who witness IPV frequently develop emotional trauma, behavioral challenges, and a higher likelihood of either perpetrating or suffering abuse later in life [35,36]. Further, IPV contributes to depression, suicidal ideation, and substance abuse, while also affecting access to education and healthcare for women and children [23]. These poor outcomes underscore the urgent need for intersectional approaches that consider gender, poverty, and health in a comprehensive and integrated manner.

### 2.1. Environmental Change and Social Vulnerability

Environmental degradation, including deforestation, pollution, and inadequate infrastructure, impacts urban livelihoods and family well-being. Urban populations in rapidly growing cities, such as Dar es Salaam, face both direct risks (e.g., floods, heat) and indirect risks (e.g., job loss, forced migration), which increase household strain [37,38]. Furthermore, resource scarcity exacerbates family conflict when household members must compete for limited essentials, such as food, water, and shelter [29]. Women bear disproportionate responsibility for resource provision, making them especially vulnerable when environmental stress escalates [39]. Additionally, displacement due to environmental change often disrupts support networks, leaving families more socially isolated and economically vulnerable [40].

### 2.2. Social Determinants and Intimate Partner Violence

Meta-analyses consistently show that IPV is linked to poor mental health, child maltreatment, and psychiatric disorders [41]. Witnessing IPV in childhood is a key risk factor for developing post-traumatic stress disorder (PTSD) and even changes in brain structure [42,43]. Poverty, gender inequality, and social isolation increase vulnerability, while systems of patriarchy normalize male control and limit women’s autonomy [44,45]. The ecological model explains how individual behavior is influenced by nested systems: from interpersonal relationships and community norms to broader societal structures and policies [46]. At each level, systemic inequalities can reinforce violence. Patriarchal norms, unequal access to education or employment, and the lack of responsive legal systems all contribute to IPV [12,47].

### 2.3. Theoretical Approaches

Feminist theory situates IPV within the context of power imbalances and structural inequalities. Patriarchal systems often frame women as property, justifying coercion and control within the family [48,49]. Structural violence, the embedded inequity in social institutions, also plays a role, limiting access to housing, justice, and healthcare [50]. The Pressure and Release (PAR) model can help conceptualize how risk emerges at the intersection of environmental hazard, vulnerable conditions, and inadequate capacity to respond [51]. In Dar es Salaam, this translates to how climate-related flooding, poverty, and weak urban governance interact to increase both stress and violence within families.

## 3. Methodology

We employed a qualitative research design method grounded in thematic content analysis and visual ethnographic observation to examine the intersection of environmental stressors and IPV in urban Dar es Salaam. The study emerged from field-based engagement and exploratory conversations and later evolved into a more structured qualitative inquiry. Given the complex, context-specific nature of the issues examined, including environmental precarity, resource scarcity, and household conflict, a qualitative approach was well-suited to generating nuanced, culturally grounded insights into how these dynamics intersect in everyday life.

Following this evolution, in-depth interviews were conducted with 10 women whose narratives were selected from a broader set of community-engaged conversations. Selection prioritized variation in housing conditions, experiences of environmental displacement, and access to social and support services, allowing the analysis to reflect a range of lived experiences shaped by intertwined environmental and socio-economic stressors. All interviews were conducted using trauma-informed practices consistent with the ethical framework described above.

### 3.1. Study Area

This research was conducted in selected urban neighborhoods of Dar es Salaam, Tanzania, with a specific focus on informal settlements located in Buguruni, Vingunguti, and the Msimbazi Valley. These areas were strategically chosen based on three interrelated criteria:

- High environmental vulnerability: These neighborhoods are frequently exposed to flooding, poor drainage systems, and waste overflow, making them hotspots for climate-induced displacement and infrastructural breakdown.
- Socio-economic uncertainty: Characterized by dense informal housing, high unemployment rates, and limited access to public services such as healthcare, legal aid, and sanitation.
- Recognition in existing documentation: These locations are consistently highlighted in policy reports, municipal planning data, and NGO assessments as zones of concentrated environmental and social stress [24,25,52].

In general, these neighborhoods represent a relevant microcosm for examining how urban environmental degradation intersects with socio-economic vulnerabilities to shape patterns of familial stress and IPV.

### 3.2. Data Sources and Collection

The study incorporated multiple qualitative data sources, beginning with document analysis and visual ethnographic observation, and later including in-depth interviews, to examine the interplay between environmental conditions and IPV in urban Dar es Salaam. Data was organized into the following categories:

1. Government Reports: Urban development strategies, environmental risk assessments, and national gender policy frameworks were reviewed from municipal and national agencies, including the Dar es Salaam City Council and the Ministry of Health.
2. NGO and Civil Society Publications: Reports and program data were analyzed from organizations such as Women in Law and Development in Africa (WiLDAF), Mkuki Coalition, and the Legal and Human Rights Centre (LHRC), focusing on gender-based violence, urban poverty, and environmental stress.
3. Academic Literature: Peer-reviewed studies (2013–2024) on IPV, climate change, urban resilience, and social determinants of health in Sub-Saharan Africa were consulted to contextualize and triangulate findings.
4. Visual Ethnographic Data: Field-based visual documentation included photographs of flood-prone zones (e.g., Vingunguti’s clogged drainage system, Buguruni’s waste overflow, and housing conditions in the Msimbazi Valley), used to supplement and spatially contextualize interview data.
5. Qualitative Interviews: As the study evolved into a structured qualitative inquiry, ten in-depth interviews were conducted with women in three informal urban neighborhoods of Dar es Salaam, Buguruni, Vingunguti, and Tandale through community-based referral networks. Eligibility criteria include being over 18 years of age, residing in flood-prone areas, and self-identifying as having experienced intimate partner violence. Interviews lasted approximately 45 min and followed a semi-structured format addressing environmental stressors, housing instability, coping strategies, access to services, and experiences of gender-based violence. Interviews were conducted in Kiswahili. To protect confidentiality, no audio recordings were made; detailed handwritten notes were taken and later translated into English for thematic analysis. Verbal informed consent was obtained prior to participation. Participants were informed of the voluntary nature of the study, their right to decline questions or withdraw at any time, and the confidential handling of their narratives. No identifying information was collected, and all accounts were anonymized.

**Figure 1:**
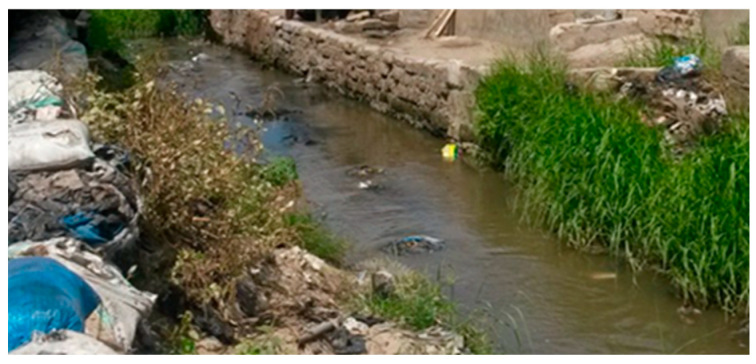
Blocked drainage along a residential area in Vingunguti, Dar es Salaam. Identified as flood-prone in urban vulnerability assessments.

**Figure 2:**
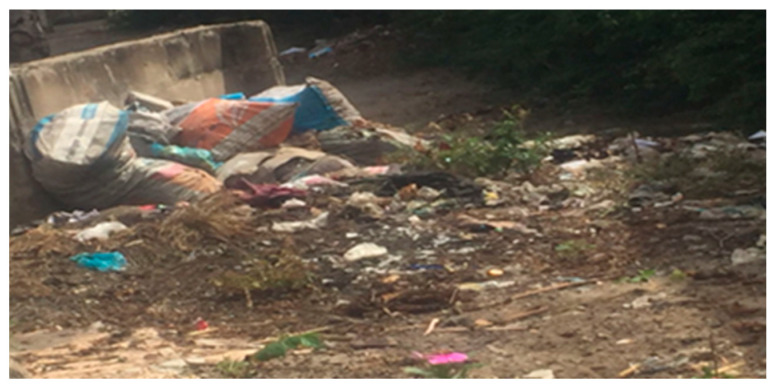
Waste overflow and informal water management were observed in Buguruni. Indicative of sanitation stress and environmental degradation.

Figure 1 and Figure 2 illustrate poor drainage in Vingunguti and unmanaged waste overflow in Buguruni, highlighting the everyday environmental stressors that increase household vulnerability in flood-prone areas of Dar es Salaam.

These visual materials complemented textual sources by providing a tangible environmental context for the lived experiences of individuals in vulnerable neighborhoods.

### 3.3. Sampling Strategy

A purposive approach guided the selection of documentary sources and participant narratives to ensure relevance to the study’s focus on environmental stressors and IPV in urban Dar es Salaam. Selection emphasized thematic alignment and variation in environmental exposure and lived experience, rather than representativeness, consistent with the study’s qualitative aims. Two distinct yet complementary sampling streams were used:

1. Documentary and Visual Sources

Documentary and visual sources were selected using predefined criteria: (1) relevance to IPV and urban vulnerability in Tanzania; (2) publication date between 2013 and 2024; (3) public or civil society origin; and (4) exclusion of images containing identifiable individuals. Sources included NGO reports, municipal planning documents, and field photography from flood-prone neighborhoods. Other criteria included:

- Direct relevance to urban Dar es Salaam, particularly informal settlements such as Buguruni, Vingunguti, Kigogo, and the Msimbazi Valley
- Topics focus on IPV, environmental degradation, urban poverty, or social vulnerability
- Publication date between 2013 and 2024 to ensure contemporary relevance
- Inclusion of visual ethnographic material (e.g., maps, photographs, spatial assessments) or community-based perspectives
- Balance between policy-level and grassroots insights, incorporating both institutional reports and civil society documentation

This approach allowed for triangulation across policy, academic, NGO, and lived-experience perspectives.

2. Participant Interviews

Ten women living in environmentally vulnerable areas of informal settlements in urban Dar es Salaam who self-identified as having experienced IPV participated in in-depth interviews as the study evolved into a structured qualitative inquiry. Participants were engaged through community-based referral networks rather than through formal recruitment, reflecting the field’s relational and context-specific nature. Selection emphasized variation in age, housing conditions, displacement experiences, and economic circumstances in order to capture a range of lived experiences shaped by environmental and socio-economic stressors.

This approach prioritized depth and contextual richness over representativeness, consistent with the study’s qualitative aims and the ethical framework described above. The analysis followed a multi-step qualitative process, incorporating both textual and visual data. As outlined in Table 1, this approach applied ecological and vulnerability frameworks to categorize emerging themes and triangulate findings across diverse data sources.

### 3.4. Data Analysis

**Table 1:** Overview of the data analysis process, including steps taken to code, analyze, validate, and interpret textual and visual data using ecological and vulnerability frameworks.

Step	Description	Framework for Categorization
1. Textual Coding	Coding of written sources to identify dominant and sub-themes related to environmental stress, IPV, gendered vulnerability, and coping mechanisms.	Ecological levels (individual, household, community, societal)
2. Visual Analysis	Analysis of photographic and video data to detect physical and symbolic indicators of urban stress, gender roles, and spatial inequality.	Dimensions of vulnerability (immediate pressures, systemic inequalities)
3. Cross-Validation	Comparison of findings across data types (e.g., NGO reports vs. photographic evidence) to confirm key patterns and contextual accuracy.	Coping strategies (adaptive, maladaptive, unsustainable)
4. Interpretive Synthesis	Integration of findings using the ecological framework and the Pressure and Release (PAR) model to situate results within wider socio-environmental systems.	Combined categorization across ecological levels, vulnerabilities, and coping strategies

### 3.5. Ethical Considerations

The study began with field-based exploratory conversations and later developed into a structured qualitative inquiry involving interviews with 10 women in informal urban settlements in Dar es Salaam. At the time of initial engagement, the project was not submitted to the IRB approval because the conversations were not initially conceived as human subjects’ research. Following the decision to analyze these narratives for scholarly publication, the study underwent ethical review by a U.S. Institutional Review Board (California State University, San Bernardino), which assessed the use of previously collected qualitative data as secondary data for compliance with ethical requirements, minimal risk, and appropriateness for publication. The IRB approved the use of these data and recommended transparency regarding the timing and nature of ethical review, affirming that narratives collected with verbal informed consent, minimal risk, and participant anonymity may remain appropriate for publication when clearly disclosed. The study adhered to the core ethical principles of the Declaration of Helsinki, U.S. federal regulations (45 CFR 46), and international best practices for research involving vulnerable populations. Participation was voluntary, verbal informed consent was obtained, and no identifying information was collected. Given cultural sensitivities, variable literacy levels, and concerns about institutional reprisal in the study context, oral consent was ethically justified and prioritized over written documentation. This approach is consistent with WHO and Council for International Organizations of Medical Sciences (CIOMS) guidance for research conducted in low-resource settings. By providing this explanation, we aim to ensure transparency and to offer a practical example of ethical research design in complex field environments where real-time ethical responsiveness may be required.

## 4. Results

This section presents the thematic findings from the content and visual analysis of environmental stressors, IPV, and related vulnerabilities in urban Dar es Salaam. The results underscore the complex interplay between ecological stress, social determinants of health, and IPV, as interpreted through ecological systems and vulnerability frameworks. In neighborhoods such as Jangwani and Kigogo, recurrent flooding not only displaces families but also intensifies household tensions by destroying homes, disrupting livelihoods, and overcrowding temporary shelters. In seven out of the ten interviews, women described increased exposure to IPV during or shortly after environmental shocks, particularly flooding, job loss, or forced displacement. These experiences align with documented trends in similar contexts [53]. Similarly, in informal settlements like Vingunguti and Mbagala, water scarcity and poor sanitation, common environmental stressors, disproportionately burden women, who are often responsible for securing household water. Long hours spent queuing or walking to collect water often trigger conflict at home, especially when men perceive these delays as neglect or a challenge to their authority. As one interviewee explained:

*“Sometimes I come back late from the water well, and my husband says I’m wasting time or seeing someone else. But the queue at the well is long, and we have no tap or running water at home. He doesn’t understand, he gets angry and starts to beat me up.”*

—Interviewee #3, Vingunguti, Dar es Salaam.

Several women described being blamed by male partners for household disruptions caused by curfews or food shortages, with accusations often rooted in expectations about women’s roles in provisioning the family. These patterns reveal how IPV in urban Dar es Salaam is not merely a product of individual or relational factors, but is deeply shaped by environmental hardship, economic marginalization, and systemic gender inequalities.

### 4.1. Impact of Resource Scarcity on Household Stress

**Figure 3:**
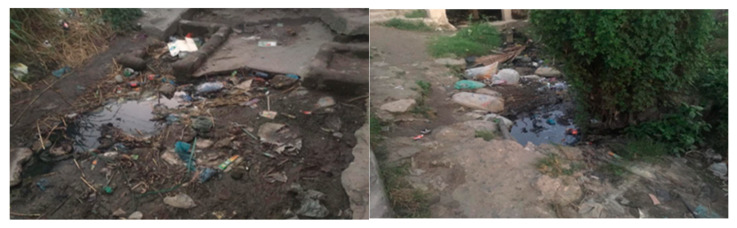
In Vingunguti, photos of blocked drains and stagnant water visually signified infrastructural neglect, aligning with reports of increased household conflict during rainy seasons.

Data shows that water scarcity, food insecurity, and inconsistent electricity supply heightened stress within families, particularly in informal settlements. The burden of sourcing these essential resources is typically shouldered by women, increasing emotional and physical tension, occasionally escalating into IPV [29,52]. Building on the conditions shown in Figure 1 and Figure 2, Figure 3 further illustrates how persistent stagnant water and blocked drains in Vingunguti reflect infrastructural neglect, a factor resident often cite as contributing to rising household tensions during the rainy season.

Household interviews cited in secondary sources confirm that when women are unable to meet daily domestic needs such as preparing food, collecting clean water, or maintaining basic hygiene, blame, emotional abuse, and sometimes physical punishment from male partners often follow. As one woman from Buguruni described:

*“That day we had no money for charcoal, and I couldn’t cook. When he came home and found no food, he yelled at me in front of the children and neighbors, then slapped me several times across the face. He said I was useless, even though he knows I tried everything.”*

—Interviewee #6, Buguruni, Dar es Salaam.

These incidents expose entrenched gendered power dynamics that are further reinforced by structural deprivation and poverty. In Dar es Salaam, particularly in low-income neighborhoods like Tandale, Buguruni, Kigogo, and Vingunguti, these dynamics are especially pronounced.

Additionally, a report by CARE International on gender and urban resilience in East Africa found that women living in flood-prone informal settlements in Dar es Salaam experienced heightened exposure to IPV during periods of environmental stress. One interviewee from Tandale reported being assaulted by her husband after returning home late from a flooded market where she had attempted, unsuccessfully, to restock her food supplies for cooking. Despite the delay being caused by infrastructure breakdown, she was blamed for “neglecting” her duties as a wife [54].

Similarly, WiLDAF-Tanzania documented testimonies from women in Mburahati and Temeke who faced violent backlash when they were unable to provide food due to sudden price hikes or failed informal earnings [52]. In several cases, women had to resort to borrowing food or cooking with alternative fuels, such as charcoal, when gas was unavailable, leading to conflict with partners who interpreted such actions as signs of incompetence or disrespect.

The Tanzania Demographic and Health Survey (TDHS), published by the Ministry of Health and supported by USAID and the DHS Program, corroborates these qualitative findings with quantitative data: 40% of urban women reported experiencing physical or sexual violence from an intimate partner, and a significant proportion of these incidents were associated with economic stressors and unmet household needs [55,56].

Moreover, Amnesty International reported that during the COVID-19 lockdowns, when access to water and food became even more constrained, women in Dar es Salaam’s densely populated areas were disproportionately blamed for the consequences of state-imposed curfews and market closures. Several women reported that their partners accused them of “failing their families,” despite circumstances being completely beyond their control.

These examples clearly illustrate how IPV in urban Dar es Salaam is both a symptom and a consequence of deeper structural issues such as poverty, inadequate infrastructure, gendered expectations, and a lack of state support for poor urban communities. For the most part, women bear the brunt of these failures, often absorbing the social and emotional consequences of environmental and economic shocks within the household.

### 4.2. Climate Change and Increased Intimate Partner Violence

Several reports have documented a clear connection between climate-related disasters, particularly floods and droughts, and heightened risks of IPV in vulnerable urban communities. Disasters in Dar es Salaam not only disrupt employment and housing but also trigger cascading effects, including economic insecurity, mental distress, and increased household conflict [56,57]. The loss of control and stability caused by these environmental shocks often amplifies gendered tensions within households, where pre-existing structural inequalities render women disproportionately vulnerable, heightening the risk of IPV.

A key example is the Jangwani Basin Upgrading Project, which was designed to mitigate flooding in central Dar es Salaam but resulted in the relocation of thousands of households from flood-prone informal settlements such as Hananasif and Kigogo. According to the World Bank’s Tanzania Urban Resilience Program and reporting from Amnesty International, many families were relocated into transitional housing or poorly planned peripheral settlements without adequate social or economic support systems, such as grocery stores and pharmacies [24,26]. These relocations fractured extended kinship networks, weakened informal safety nets, and increased isolation conditions that, according to CARE International, heightened the risk of IPV [54]. Further, women in relocated households reported that their partners, frustrated by unemployment or loss of social status, became more controlling and aggressive in the aftermath of displacement.

Moreover, loss of income during these climate crises was particularly detrimental in households already marked by economic precarity. For instance, in flood-affected neighborhoods like Tandale and Vingunguti, many men who relied on daily wage labor or informal vending lost their livelihoods when markets were submerged or transportation routes were cut off. One participant described how flooding-related job loss, alcohol use, and forced relocation contributed to escalating violence and social isolation:

*“He lost his job during the floods and started drinking. After that, everything changed at home, and the beatings increased. Besides, I used to go to my neighbors for help, but now I’m far and don’t know anyone here…”*

—Interviewee #6, Vingunguti, Dar es Salaam.

This economic disempowerment, as highlighted by WiLDAF-Tanzania [52], often led to increased alcohol consumption and expressions of frustration through verbal or physical abuse toward their partners. Simultaneously, women became more dependent on these same partners for basic survival needs, further entrenching power imbalances and limiting their ability to seek help or leave violent relationships.

Furthermore, [57] found that the psychological toll of climate disasters, particularly among men, was rarely addressed through formal mental health support, leading to unresolved trauma and aggression that frequently played out in the domestic sphere [57]. This reinforces the need for integrated urban planning that not only addresses environmental risks but also anticipates and mitigates the social consequences of climate-induced displacement and economic shock.

### 4.3. Displacement and Family Dynamics

Environmental displacement due to floods or informal eviction in urban Dar es Salaam has frequently resulted in highly stressful and precarious living arrangements. Displaced households often found themselves in overcrowded temporary shelters, schools, or shared accommodations with extended family members, conditions that introduced new social frictions and exacerbated existing gender inequalities. These stressors were particularly acute among displaced women. One interviewee described how relocation disrupted household dynamics and increased her vulnerability:

*“I had no say in anything after we moved. I depended on my husband for everything, and that made me feel trapped...”*

—Interviewee #2, Msimbazi Valley, Dar es Salaam.

Additionally, these dislocations had several interlinked consequences:

- Disruption of traditional family roles: In many cases, displacement challenged normative gender dynamics and household hierarchies. Men who lost their roles as primary providers due to the destruction of income sources such as kiosks, market stalls, or transport work often experienced a sense of disempowerment. According to CARE International, this perceived loss of authority sometimes led to increased controlling behavior or violence toward partners, particularly when women attempted to take on more public or income-generating roles [54].
- Erosion of women’s privacy and autonomy: In temporary shelters in areas like Hananasif and Jangwani, women often shared small rooms with multiple family members, including in-laws, making it nearly impossible to maintain personal boundaries or secure spaces for self-care or confidential communication. Reports from Amnesty International and WiLDAF-Tanzania document several cases where women expressed feeling unsafe, exposed, and constantly surveilled, especially by male relatives of host families [24,52]. This loss of autonomy not only increased psychological distress but also made it harder for survivors of IPV to report abuse or seek help.
- Tension between host and displaced families: Overcrowded housing frequently generated conflict between host and displaced families, especially when resources such as food, water, or sanitation facilities were scarce. A study by Bunce et al (2010) [56] found that in flood-displaced communities in coastal areas, women were often scapegoated for “overburdening” the household, leading to strained relationships and increased emotional or physical abuse within the extended family setting [58].

For example, following the 2019 floods in the Msimbazi basin, several families from Jangwani were temporarily relocated to public school buildings and then informally absorbed by relatives in surrounding neighborhoods. According to interviews cited in the Tanzania Urban Resilience Program [26], women in these settings reported feeling both socially isolated and blamed for the family’s displacement. In one case from Kigogo, a woman who was sheltering with her husband’s brother’s family faced verbal abuse from her sister-in-law for “failing to manage her household,” even though the loss of their home was due to climate-related flooding. Her husband, under stress from unemployment and loss of status, reportedly turned more violent during this period, blaming her for “bringing shame” to the family.

One participant recounted how losing her home during flooding forced her into overcrowded housing and limited her ability to seek safety:

*“When the floods came, everything was gone. I had to move in with my husband’s brother’s family. It was crowded, and I had no privacy. My husband was always angry, and one night, he blamed me for making us poor and hit me. I couldn’t say anything because we had nowhere else to go.”*

—Interviewee #7, Kigogo, Dar es Salaam

These examples illustrate that environmental displacement is not just a logistical or infrastructural challenge; it has deep social and gender consequences. The breakdown of normal household routines, combined with overcrowding and loss of control over space and resources, can significantly elevate IPV risks and deepen women’s vulnerability in crisis contexts.

### 4.4. Role of Social Determinants of Health

Our findings confirm that poverty, lack of education, limited access to healthcare, and entrenched gender inequality significantly mediate the relationship between environmental stressors and IPV in urban Dar es Salaam. These intersecting structural factors not only increase the risk of IPV but also diminish the capacity of women to seek help or escape abusive situations, especially in informal settlements. Women with low levels of literacy or those lacking formal education are often unaware of their legal rights or available support systems. As one interviewee from Buguruni noted:

*“I don’t know where to go. I have heard people talk about Social Welfare women’s desks in our district, but I’ve never seen one. I can’t read, so I rely on what others tell me.”*

—Interviewee #4, Buguruni, Dar es Salaam.

These patterns underscore that a lack of education contributes to women from these affected neighborhoods relying on male partners for decision-making and access to information, reinforcing the fact that power imbalances within the household persist.

Women with limited literacy or basic education were frequently unaware of their legal rights or available support services. A qualitative study by WiLDAF-Tanzania (2020) found that women in areas like Buguruni, Kigogo, and Tandale were often unaware of the existence of gender desks at police stations or the legal channels for reporting abuse [52]. Some even feared that reporting partner violence would lead to further backlash or social stigma due to cultural norms discouraging women from “exposing family matters.” These barriers were particularly acute among women who had not completed primary school or who migrated from rural areas with little exposure to formal institutions [52].

Households in unplanned settlements such as Vingunguti, Kigogo, and parts of Temeke faced geographical and infrastructural isolation. Clinics were distant and often under-resourced, and police posts were few and ill-equipped to handle gender-based violence cases. According to the Tanzania Urban Resilience Program (World Bank, 2020) [26], many women in these neighborhoods reported needing to walk long distances to access basic health services or report instances of IPV. In one example, a woman from Kigogo stated that she had to travel over 5 km to reach a clinic after a violent incident, only to be referred to a distant hospital with no transport assistance. This not only discouraged women from seeking help but also reinforced a sense of helplessness, given the limited resources available. For example, an interviewee living around the Kigogo neighborhood described significant challenges accessing healthcare, police services, or psychosocial support. Clinics and police posts were either too far, under-resourced, or unresponsive to IPV-related cases:

*“There’s a clinic nearby, but they don’t take you seriously if you say your husband beat you. They just tell you to solve it at home.”*

—Interviewee #2, Kigogo

Further, men interviewed in these same neighborhoods frequently expressed frustration with chronic unemployment or underemployment, especially during environmental stressors such as floods, market closures, or infrastructure collapse. As documented in CARE International’s Dar es Salaam case study, several men who previously worked as informal traders or construction laborers lost their income during the 2019 seasonal floods. Some described feeling emasculated or “useless” because they were unable to provide for their families. These emotional and social pressures, when coupled with substance use and poor mental health support, often translate into controlling or violent behavior toward their partners, which one respondent described as “taking back control at home” [54].

This dynamic, where male disempowerment intersects with female dependency in fragile socio-environmental contexts, creates a volatile domestic environment where IPV becomes both a symptom of broader structural failures and a coping mechanism for deeply ingrained gender hierarchies. These patterns underscore the pressing need for integrated approaches that combine climate-resilient urban planning with gender-transformative programming to address the root causes of IPV and strengthen support systems.

### 4.5. Coping Mechanisms

Survivors of IPV and environmental stress in Dar es Salaam adopted a range of both adaptive and maladaptive coping strategies in response to the intersecting pressures of poverty, insecurity, and gender-based violence. Many women sought help from relatives or extended family members, which provided short-term emotional and material relief, but in several cases, this strained kinship ties or inadvertently enabled continued abuse, particularly when family members prioritized household unity over individual safety [53]. Others coped through avoidance and silence, choosing not to confront abusive partners to “keep the peace” or protect children, a tactic that may reduce immediate conflict but often deepens psychological trauma and normalizes the cycle of violence over time. As one interviewee shared:

*“I went to stay with my sister after he beat me, but she told me to go back and ‘be patient.’ She said every marriage has problems. Since then, I just stay quiet to avoid more trouble.”*

—Interviewee #8, Tandale, Dar es Salaam.

Furthermore, some women turned to informal labor, such as petty trading or domestic work, to regain a sense of agency and earn a living. While this strategy offered temporary economic relief, it also exposed them to exploitative labor conditions, long hours, and the risk of harassment, especially in unregulated markets and in employers’ homes [59]. In more extreme cases, survivors reported resorting to substance use or transactional sex to cope with emotional stress or meet basic needs [54]. While these behaviors provided immediate escape or financial support, they significantly increased vulnerability to further violence, health risks, and social stigma. These patterns highlight the urgent need for trauma-informed, multi-sectoral support systems that can offer sustainable alternatives and long-term safety for IPV survivors facing environmental and social hardships [53,59]. As shown in Table 2 below, women in Dar es Salaam informal settlements adopt a range of both adaptive and maladaptive coping strategies in response to intersecting environmental, economic, and interpersonal pressures. While seeking help from relatives may offer short-term relief, it can strain kinship ties over time. Other strategies, such as avoidance or engaging in informal labor, carry long-term risks including trauma, exploitation, or continued exposure to violence.

The placement of violence within the exosystem reflects how external disruptions, such as flooding, eviction, or policy failures, can create the conditions for IPV escalation. At the chronosystem level, we acknowledge that intergenerational trauma and long-term structural neglect also shape family dynamics and responses to stress.

### 4.6. Key Themes and Framework Alignment

A multi-level thematic interpretation of the findings was conducted using the Ecological Systems Theory and the Vulnerability Framework to understand how environmental stressors interact with IPV in urban Dar es Salaam. At the microsystem level, resource scarcity, such as a lack of water or food, creates immediate household pressure, often leading to interpersonal conflict. The mesosystem highlighted how displacement and disrupted gender roles weakened social support networks, increasing emotional and financial strain.

At the exosystem level, climate shocks like floods acted as triggering events for IPV, particularly when followed by loss of income or housing. The macrosystem revealed how structural inequalities such as poverty, gender norms, and limited access to services reinforce cycles of violence. Lastly, the chronosystem revealed how survivors adopted coping mechanisms over time; however, many of these strategies (e.g., silence, transactional labor) provided only short-term relief while deepening their long-term vulnerability. As Table 3 demonstrates, themes emerging from the data reflect how different levels of environmental and social pressure influence experiences of IPV. These include household tensions stemming from resource scarcity, disruptions to support systems, and long-term structural inequalities.

**Figure 4:**
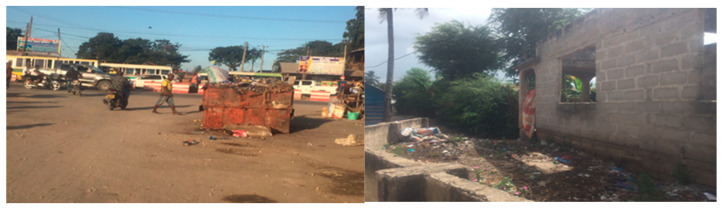
The overflowing trash in Buguruni symbolizes systemic neglect and poses an environmental hazard, contributing to stress, disease, and a decreased quality of life, especially among women responsible for household sanitation.

**Figure 5:**
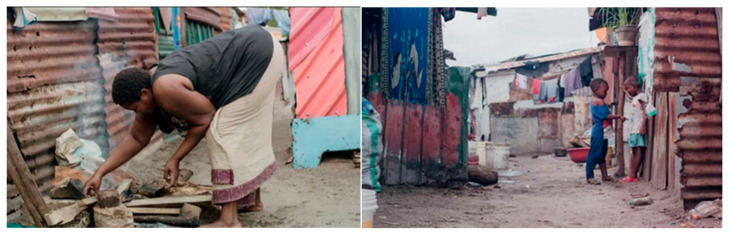
A woman cooks over a firewood stove with children nearby in a flood-prone area, exemplifying how care burdens, environmental exposure, and poverty intersect in daily life.

Table 3 Thematic interpretation of environmental stressors, social vulnerability, and IPV across different levels of influence. Further, Figure 4 and Figure 5 below show environmental degradation, such as overflowing waste and reliance on open-firewood cooking, in flood-prone areas, illustrating how systemic neglect, poverty, and gendered care burdens converge to increase stress and health risks for women in urban Dar es Salaam.

### 4.7. Visual Evidence and Urban Context

## 5. Discussion

The findings of this study reveal a deeply interconnected, multi-layered relationship among environmental stressors, social determinants of health, and IPV in urban Dar es Salaam. Anchored in a socio-ecological and vulnerability framework, the analysis demonstrates how systemic environmental degradation, recurring climate shocks, forced displacement, and entrenched social inequalities converge to heighten women’s exposure to IPV, especially in low-income, informal settlements. Rather than acting in isolation, these factors interact across ecological levels to shape survivors lived experiences. At the household level, resource scarcity and economic stress inflame existing gendered power imbalances; at the community level, displacement and weakened social networks reduce safety nets and support systems. Consequently, at the structural level, persistent poverty, inadequate infrastructure, and limited access to justice compound women’s vulnerability. These dynamics underscore the urgent need for multi-sectoral interventions that address not only interpersonal violence but also the broader environmental and systemic conditions that perpetuate it.

### 5.1. The Interplay of Environmental Stressors and Intimate Partner Violence

Environmental degradation, particularly in flood-prone urban neighborhoods, affects not only infrastructure and public health but also reshapes household dynamics. When women are primarily responsible for water, food, and energy procurement, resource scarcity transforms into gendered stress and emotional or physical violence. The climate-induced loss of income, food, and shelter increases male frustration and may result in violent behavior toward partners, as other studies have documented in post-disaster contexts [60,61].

The feminist ecological model used in this research emphasizes that violence must be understood in relation to broader systems of gendered power and environmental inequality. Environmental stressors act not just as stress multipliers, but as structural triggers that expose or amplify pre-existing patriarchal power imbalances [62].

### 5.2. Implications for Urban Planning

This study highlights a critical gap in current urban planning in Dar es Salaam: the failure to integrate climate adaptation with social protection and gender equity. Uncontrolled urbanization has led to informal settlements that are environmentally hazardous and socially fragile, particularly for women and children. Basic services, such as water, sanitation, waste management, and affordable housing, are often unavailable or inaccessible in these areas. 

**Figure 6:**
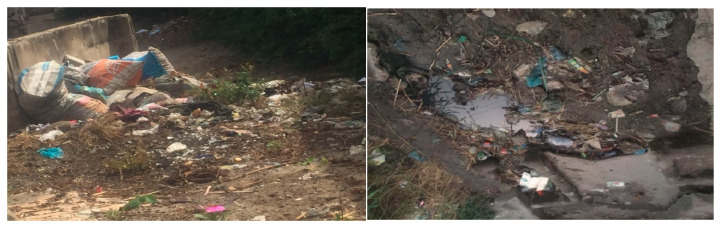
Visual evidence: The accumulation of trash outside homes underscores the environmental neglect that fuels health risks, household tension, and violence.

As depicted in Figure 6, the accumulation of trash outside homes reflects ongoing environmental neglect, which contributes to health hazards, household tension, and potential triggers for intimate partner violence in low-income urban areas.

Urban planning must take a resilience-building approach, including:

- Environmental risk reduction (e.g., flood mitigation, drainage improvement)
- Inclusion of gender-sensitive indicators in infrastructure investment
- Participatory governance that includes women’s voices from informal settlements

### 5.3. Policy Recommendations

The results of this research call for an integrated, multisectoral policy response to reduce both IPV and environmental vulnerability:

1. Community-based social support systems: Establish neighborhood-level safe spaces for counseling, conflict resolution, and psychosocial services.
2. Women’s economic empowerment: Expand access to vocational training, microcredit, and employment opportunities in sustainable industries.
3. Access to services in informal settlements: Ensure provision of WASH (water, sanitation, and hygiene), healthcare, and legal services in underserved neighborhoods.
4. Cultural transformation: Fund educational campaigns that challenge harmful gender norms, encourage shared domestic responsibilities, and de-normalize IPV.
5. Inclusive environmental policy: Align disaster preparedness and urban environmental management plans with IPV prevention programs.
6. Mental health integration: Develop trauma-informed services to support women and children affected by both IPV and climate-related displacement.

These recommendations align with international best practices for urban resilience, public health, and gender justice [2,7,63].

### 5.4. Study Limitations

While this study sheds light on the challenges women faces due to environmental stressors, it has several limitations. First, the issue of generalizability cannot be ignored. While the findings provide critical insight into the intersection of environmental stressors, gender inequality, and IPV in urban Dar es Salaam, they are context-specific and not automatically transferable to other urban centers in the Global South. Cities may differ significantly in terms of governance systems, infrastructure, cultural norms, and socioeconomic conditions, all of which influence how households perceive vulnerability and resilience. Comparative studies highlight that even when urban areas share similar challenges, the lived realities and policy responses can diverge considerably [64]. Therefore, caution is needed in applying these results more broadly without further cross-contextual analysis. Secondly, another limitation of this study is its temporal scope, as some findings may be influenced by post-COVID-19 dynamics that are not fully captured in the source documents analyzed. The pandemic significantly altered economic precarity, household dynamics, and patterns of gender-based violence in many urban settings, introducing new vulnerabilities while exacerbating existing inequalities. Emerging research suggests that COVID-19 lockdowns, disruptions to services, and long-term socioeconomic impacts reshaped the landscape of IPV, with women in low- and middle-income countries facing heightened risks and reduced access to support systems [65]. As such, interpretations of the current findings should be made with caution, acknowledging the potential influence of these post-pandemic shifts. Given these limitations, we recommend that future research incorporate longitudinal ethnographic studies to enrich the emerging field of environmental IPV.

### 5.5. Future Research Directions

Further studies should:

- Examine the mental health implications of dual exposure to IPV and environmental shocks.
- Explore child outcomes, especially regarding intergenerational trauma linked to IPV.
- Analyze the role of masculinity, unemployment, and social identity under climate stress.
- Develop place-based intervention models linking environmental planning with violence prevention and gender empowerment.

An interdisciplinary approach drawing from urban planning, gender studies, public health, and climate science is essential to produce actionable knowledge for policy and practice [66,67].

### 5.6. The Role of Urban Resilience

The study highlights the need to rethink urban resilience beyond infrastructure. True resilience must include:

- Social resilience: Strengthening relationships, mental health, and gender equity.
- Economic resilience: Ensuring employment and food security.
- Environmental resilience: Protecting urban populations from climate risk.

Cities like Dar es Salaam must recognize that environmental justice and gender justice are inextricably linked. Urban planning efforts, especially under climate pressure, must be designed not only for physical infrastructure but also to mitigate social vulnerability, including gender-based violence. While IPV is never a justifiable response, environments that leave women isolated and unsupported increased risk. Therefore, coordinated efforts must target both prevention of perpetration and the systems that allow it to persist.

### 5.7. Towards Integrated Development

Policymakers and urban planners must adopt integrated, intersectional frameworks that address environmental stress, social inequality, and IPV simultaneously. Resilience strategies that ignore the lived experiences of marginalized groups, particularly women in informal settlements, risk deepening rather than solving urban challenges. As emphasized in the Sustainable Development Goals (SDGs), especially SDG 5 (Gender Equality) and SDG 11 (Sustainable Cities and Communities), cross-sector collaboration is vital [68,69,70]. The recommendations outlined in this study can help guide:

- Urban policy development
- Gender-responsive disaster risk reduction in Tanzania
- Community-based health and safety programming

### 5.8. Call to Action

In closing, our research affirms that domestic violence, particularly IPV in urban Dar es Salaam, cannot be understood or addressed in isolation. It is not merely a private or interpersonal issue but a manifestation of deeper, structural failures in governance, environmental planning, and social protection systems. From inadequate infrastructure and climate vulnerability to weak legal enforcement and persistent gender inequality, the conditions that enable IPV are embedded in the urban fabric itself. The normalization of violence is sustained not just by individual attitudes but by systemic neglect [71,72].

Combating IPV, therefore, requires more than awareness campaigns or reactive legal frameworks; it demands a transformation of urban systems that consistently fail to protect their most vulnerable residents. This includes investing in safe and equitable housing, expanding access to essential services such as healthcare and legal aid, and integrating gender-sensitive planning into climate adaptation and disaster response strategies [54,71,72].

If urban resilience is to be truly meaningful, it must extend beyond the ability to withstand floods, droughts, and heatwaves. Further, urban resilience must also include the capacity to confront and prevent the silent crisis of violence that unfolds behind closed doors, violence that is every bit as destructive as any environmental disaster. A resilient city is not just one that survives climate shocks, but one that ensures safety, dignity, and justice for all its inhabitants, especially for women and girls navigating the dual vulnerabilities of environmental precarity and IPV.

## 6. Conclusions

This study offers a nuanced examination of the relationship between environmental stressors, social determinants of health, and IPV in urban areas, with a focus on Dar es Salaam, Tanzania. By integrating visual ethnography, interviews, thematic analysis, and theoretical frameworks, particularly the ecological model and feminist perspectives, we find compelling evidence that resource scarcity, climate-related displacement, and urban neglect are not merely environmental or economic issues but deeply gendered social vulnerabilities. Environmental stressors such as floods, heat, and displacement exacerbate existing household tensions and power dynamics. These stressors are filtered through poverty, lack of education, unemployment, and gender inequality, making vulnerable populations (especially women) more susceptible to intimate partner violence.

## Figures and Tables

**Table 2:** Coping strategies adopted by survivors of IPV and environmental stress in urban Dar es Salaam, with short- and long-term consequences. Data synthesized from field interviews and secondary sources.

Coping Strategy	Short-Term Effect	Long-Term Implication
Seeking help from relatives	Provides temporary relief	May strain kinship ties or enable abuse
Avoidance and silence	Reduces confrontation	Deepens trauma and normalizes abuse
Engaging in informal labor	Offers economic relief	May involve exploitative conditions
Substance use or transactional sex	Immediate escape or income	Heightens risk of violence and stigma

**Table 3:** Thematic Interpretation Through Theoretical Frameworks.

Theme	Ecological Level	Vulnerability Dimension	Insight
Resource scarcity and household tension	Microsystem	Immediate pressure	Translate into interpersonal conflict
Displacement and role disruption	Mesosystem	Disruption of support networks	Gendered roles challenged
Climate shocks and violence	Exosystem	Hazards/event shocks	Trigger IPV post-disaster
Structural inequality	Macrosystem	Root causes	Inequities reinforce cycles of abuse
Coping mechanisms	Chronosystem	Temporal resilience	Short-term fixes deepen long-term risk

## Data Availability

The qualitative data collected and analyzed during this study, including interview transcripts and field notes, are not publicly available due to privacy concerns and ethical restrictions. The data contains sensitive personal narratives related to intimate partner violence and environmental stress, and participants were assured confidentiality and anonymity as a condition of participation. Sharing this data would risk re-identification and potential harm. Researchers may request further methodological details by contacting the corresponding author, subject to institutional ethical guidelines.
